# Biodistribution and dosimetry of the carbon-11 labelled PARP1-selective PET radiopharmaceutical [^11^C]AZ14193391

**DOI:** 10.1186/s13550-026-01481-1

**Published:** 2026-08-19

**Authors:** Anders Törnblom, Cecilia He, Martin Bolin, Antonios Tzortzakakis, Josefin Fernebro, Chunde Li, Susanne Fridsten, Renske Altena, Rimma Axelsson, Jonathan Siikanen, Cecilia Hindorf

**Affiliations:** 1 Department of Nuclear Medicine and Medical Physics Nuclear Medicine, Karolinska University Hospital, Stockholm, Sweden; 2 Division of Molecular Medicine and Surgery (MMK), Karolinska Institutet, Stockholm, Sweden; 3 Division of Radiology Department for Clinical Science Intervention and Technology (CLINTEC), Karolinska Institutet, Stockholm, Sweden; 4 Centre for Psychiatry Research Department of Clinical Neuroscience, Karolinska Institutet & Stockholm Health Care Services Region, Stockholm, Sweden; 5 Department of Nuclear Medicine and Medical Physics Theranostics Trial Center, Karolinska University Hospital, Stockholm, Sweden; 6 Department of Oncology-Pathology, Karolinska Institutet, Stockholm, Sweden; 7 Section of Gynecological Oncology, Karolinska University Hospital, Stockholm, Sweden; 8 Department of Oncology, Södersjukhuset, Stockholm, Sweden; 9 Department of Clinical Science and Education, Karolinska Institutet Södersjukhuset, Stockholm, Sweden

**Keywords:** [^11^C]AZ14193391, PET, PARP1, PARP-inhibitor, Radiation dosimetry, Biodistribution, TIAC, Parametric bootstrap

## Abstract

**Background:**

Poly (ADP-ribose) polymerase-1 (PARP1) inhibitors are used for treatment of advanced breast, ovarian, and prostate cancer with germline or somatic *BRCA1/2* mutations. However, not all patients respond to these drugs, and therapy-predictive biomarkers are needed to optimise the benefit-risk balance. The ^11^C-labelled PARP1-selective radiopharmaceutical [^11^C]AZ14193391 has emerged as a promising radioligand to identify eligible patients. The aim of this study was to investigate the safety and to determine the biodistribution and dosimetry of [^11^C]AZ14193391. A secondary aim was to investigate the parametric bootstrap method to determine time-integrated activity coefficients (TIACs), absorbed doses, and effective dose.

**Results:**

Ten participants (5 breast, 3 ovarian, 2 prostate cancer) were injected with 5.7 MBq/kg [^11^C]AZ14193391. Participants underwent five positron emission tomography (PET) acquisitions, ranging from the base of the skull to the proximal femur, at approximately 0, 10, 20, 30, and 45 min post-injection. Participants were monitored for changes in vital signs and were contacted 24 h after injection. No adverse events occurred. Visual evaluation of PET images showed high activity concentrations in the gastrointestinal tract, liver, kidneys, red marrow, pancreas, salivary glands, and spleen. Organs were segmented on a low-dose computed tomography image with an automatic segmentation tool (TotalSegmentator extension in 3D Slicer). The segmentations were reviewed and corrected based on the PET images. Time-activity curve (TAC) fits were determined with two TAC fit methods: 1) individual and 2) parametric bootstrap, to calculate TIACs. Absorbed doses and effective dose were determined with IDAC-Dose 2.1 from eleven source organs. Mean effective dose was estimated to be 5.0 (SD = 0.3) µSv/MBq with individual TAC fits and 5.0 (SE = 0.1) µSv/MBq with parametric bootstrap TAC fits. The organs receiving the highest mean absorbed doses per injected activity were liver, kidneys, and spleen. Gallbladder, red marrow, salivary glands, and spleen showed continued accumulation during the PET acquisitions.

**Conclusions:**

Imaging with [^11^C]AZ14193391 is feasible and safe. Our results suggest that the radiation-induced risk is low, warranting further studies to evaluate the potential benefits of this diagnostic approach. The parametric bootstrap method estimated the population variability in the effective dose, but not in the TIACs.

**Clinical Trial Registry:**

Ongoing recruitment Human Pharmacology (phase I) trial in Sweden including cancer patients in the age range (18–64) years. Sponsor: Karolinska University Hospital. https://euclinicaltrials.eu/ctis-public/view/2022-502918-87-00: Registry identifier: EU CT 2022–502918-87–00; Date of registration: 2023–08-17.

**Supplementary Information:**

The online version contains supplementary material available at 10.1186/s13550-026-01481-1.

## Background

Poly (ADP-ribose) polymerase (PARP) is a family of enzymes involved in the DNA damage response where PARP1 is the most abundant and best-characterised [1]. PARP1 can be upregulated in some cancers in response to increased DNA damage and genomic stress [2, 3]. Since PARP1 is involved in DNA repair, particularly the repair of single-strand DNA breaks, increased PARP1 expression may help tumour cells survive despite a high burden of DNA damage [3]. Inactivation of PARP leaves damaged DNA unrepaired, which can lead to apoptosis [4, 5]. PARP inhibitor (PARPi) drugs have therefore been developed as a targeted therapy for cancer. Olaparib was the first clinically approved PARPi and was initially developed to target homologous recombination deficiency (HRD) in breast and ovarian cancer patients with *BRCA1/2* mutations [6]. Since then, several cancer types have been found to have increased expression of PARP enzymes [7]. To date, the European Medicines Agency has approved the use of PARPi (olaparib) for treatment of subtypes of early and advanced ovarian, breast, prostate, endometrial, and pancreatic cancer [8].

The predictive biomarkers used today test downstream pathways and not PARP expression itself, mainly *BRCA1/2* mutations and other gene defects contributing to HRD [9]. Although *BRCA1/2* mutations and HRD status are important determinants of PARPi sensitivity, they do not fully account for the variability in patient response with response rates of approximately 30–50% [10–12]. Additionally, clinical trials have shown that patients without germline or somatic *BRCA1/2* or other HRD mutations can also benefit from PARPi [13–15], indicating that additional molecular and clinical factors contribute to treatment response. In the setting of metastatic disease, there is usually intra- and intertumoural heterogeneity among the metastases in the same patient, which means that the biopsy of a single lesion may not reflect the status of the disease in the rest of the body [16–18]. For advanced breast, ovarian, and prostate cancer, *BRCA1/2* mutations could be measured in circulating tumour DNA (ctDNA) [19–21]. The ctDNA can aid identification of patients with germline or somatic *BRCA1/2* mutations. However, ctDNA does not specify the location or the number of metastases with *BRCA1/2* mutations, which leaves a gap in therapeutic predictability. To date, no high-accuracy therapy-predictive biomarkers for PARPi have been established to optimise the benefit-risk balance.

Positron emission tomography (PET) is a non-invasive, in vivo*,* whole-body imaging modality that, combined with a PARP1-targeted radiopharmaceutical, could potentially be used to identify patients eligible for PARPi treatment and to predict their response. A theranostic approach with PARP-targeted therapeutic radiopharmaceuticals has been explored in preclinical studies [22, 23]. There are no PARP-selective diagnostic radiopharmaceuticals approved for clinical use, but several are being evaluated in preclinical and clinical research settings [24].

Novel radiopharmaceuticals require evaluation of dosimetry to estimate the associated effective dose, which is part of the justification for clinical use. A novel PARP1-selective radiopharmaceutical, [^11^C]AZ14193391, can penetrate the blood–brain barrier has the potential to facilitate patient selection for PARPi treatment [25]. Cselényi et al. (2025) presents first-in-human PET imaging with a focus on the brain. In vitro autoradiography results showed total blockade of [^11^C]AZ14193391 by olaparib in human cerebellum tissue and low non-specific binding in glioblastoma tumour tissues when co-incubated with AZD9574 (palacaparib) [26]. However, whole-body PET imaging with [^11^C]AZ14193391 to identify PARP1-positive metastases has not been investigated.

In this study, our aim was to investigate the safety, determine the biodistribution, and calculate the absorbed doses and effective dose in humans for PET imaging with [^11^C]AZ14193391. A secondary aim was to investigate the parametric bootstrap method’s ability to estimate the population variability in time-integrated activity coefficients (TIACs), absorbed doses, and effective dose.

## Methods

### Participants

This study included participants between 2023-Oct and 2025-June from an ongoing clinical trial based in Region Stockholm, Sweden. The trial’s aim is to recruit PARPi-naïve patients, part of which are scheduled for systemic PARPi treatment from three patient groups with advanced/metastatic solid tumours: 1) *HER2*-negative breast cancer independent of *BRCA1/2* status, 2) ovarian cancer independent of *BRCA1/2* status, and 3) castration-resistant prostate cancer with germline or somatic *BRCA1/2* gene mutations.

All clinical trial participants provided written informed consent. The clinical trial was approved by the Swedish Ethical Review Authority and the Swedish Medical Products Agency (Dnr: 5.1.1–2023-23981, EudraCT: 2022–502918-87–00) and conducted in accordance with the Declaration of Helsinki.

### Monitoring safety

The safety monitoring of the participants included: body temperature, heart rate, systolic and diastolic blood pressure, and respiratory rate before and after examination. Participants were contacted approximately 24 h after examination to detect any adverse events (AE).

Any clinically significant increase in the original blood pressure or heart rate value, onset of fever, dyspnoea, or skin rash was counted as an AE. A clinically significant increase in blood pressure or heart rate value is defined either as a 50% increase in the original value or values above 180/120 mmHg and > 110 beats per minute, respectively. Fever is defined as tympanic temperature above 38.5°C.

### Imaging protocol

Participants received an intravenous bolus injection of 5.7 MBq/kg body weight of [^11^C]AZ14193391 and the maximum injected protein mass was 10 µg (treatment dose of olaparib is 300 mg/day [27]). A Discovery MI PET/CT (GE Healthcare, Milwaukee, WI, USA) with 5 detector rings was used for PET and computed tomography (CT) acquisition. An attenuation-correction CT was performed prior to the PET acquisitions. Participants were imaged from the base of the skull to the proximal femur five times, starting at approximately 0, 10, 20, 30, and 45 min post injection (p.i.). All PET acquisitions were completed one hour p.i. Time per bed position of the sequential acquisitions was balanced against decay to achieve equivalent count statistics in all acquisitions. Participants did not void during imaging. After PET acquisitions, a low-dose CT for organ segmentation was performed.

All relevant PET corrections were included (time-of-flight, attenuation, scatter, normalisation, decay, and dead-time correction). PET image reconstruction was performed with block-sequential regularized expectation maximization (Q.Clear) with β = 350 and point-spread function correction.

### Segmentation

Source organs were, initially, automatically segmented on the low-dose CT using 3D Slicer’s plugin TotalSegmentator [28, 29]. TotalSegmentator facilitates user-independent segmentation. However, due to motion artefacts and partial volume effects (PVEs) [30], each segment used for dosimetry was manually corrected to cover the full PET signal in all acquisitions. For organs with high activity concentration (liver, spleen, kidneys, gallbladder contents, urinary bladder, and salivary glands), segments were defined on each PET acquisition, whereas organs with lower uptake were defined in the CT image and transferred to the PET images. If tumours and metastases were found in source organs, they were excluded from the corresponding segment, and total activity was estimated as the segment mean activity concentration (${A}_{c}$ [Bq/ml]) multiplied by the source organ volume measured on the low-dose CT. The completed segments were reported as total activity (${A}_{tot}$ [Bq]) or ${A}_{c}$ depending on the source organ. Total blood activity was estimated from aortic ${A}_{c}$ measured with a cylindrical volume of interest with a diameter of 14 mm and a height of 42 mm to reduce PVE and multiplied by a factor of 65 (women) or 75 (men) ml blood per kg body weight [31]. In skeletal tissue [^11^C]AZ14193391, like other PARP1-selective drugs, primarily binds to the red bone marrow (RM) [32]. Therefore, the activity in RM could be approximated as the total activity measured in bone segmented from the total skeletal structure. However, to minimise the interference from PVE, RM was measured in the lumbar spine (L1-L5). The EANM guidelines for bone marrow dosimetry estimate that 12.3% of RM is present in the lumbar spine [33]. ${A}_{tot,RM}$ was determined according to Eq. 1.

1 $${A}_{tot,RM}=\frac{{A}_{c,L1-L5} \times {V}_{tot,L1-L5}}{0.123}$$

where ${A}_{c,L1-L5}$ is the activity concentration in the lumbar spine and ${V}_{tot,L1-L5}$ is the lumbar spine volume measured on the low-dose CT image.

### Time-activity curves

The time-activity curves (TACs) were calculated and plotted in RStudio [34]. Source organ measurements were decay-corrected to time of injection and converted to percentage of injected activity (*%IA*).

Source organ TAC models were chosen based on two criteria: i) The model should follow the shape of the measured activity over time (is there an uptake and/or retention phase) and ii) The model should use the lowest number of fitting parameters.

Two methods for source organ TAC model fits were used: 1) Individual TAC fits and 2) Parametric bootstrap estimation, accounting for errors in activity estimates, i.e. least-squares method combined with Monte Carlo simulation. Both methods determine time-integrated activity coefficients (TIACs), organ absorbed doses, and the effective dose, but yield different types of uncertainties. The individual TAC method reflects the variability in the sampled cohort, and the parametric bootstrap method estimates the uncertainty of the mean in the sampled cohort.

#### Individual TAC fit

For individual TAC fits, identical models to the parametric bootstrap models were applied for respective source organ, except for: i) Linear fits with non-significant slope (*p* > 0.05) where a constant value was assigned based on the mean of measured activity across acquisitions for each study participant, and ii) Non-linear fits that did not converge where the model was adjusted based on the TAC model criteria listed in Time-activity curves section for each study participant.

#### Parametric bootstrap TAC fit

To estimate the uncertainty in the mean of the cohort, the parametric bootstrap method (iterative random resampling) was used to determine TAC model parameters, i.e. simulation of new measurements based on the sampled cohort.

Source organ measurements were binned into one of five time-post-injection (*TPI*) intervals, i.e. acquisition 1, 2, 3, 4, and 5. For each bin *j,* the mean (${\overline{TPI} }_{j}$ and $\overline{\%IA}_{j}$) and the respective standard errors (${SE}_{TPI,j}$ and ${SE}_{\%IA,j}$) were calculated. Each iteration *i* (range [1, *k*]) generated one random data point for each bin *j* (range [1, 5]) as

2 $${{TPI}_{i,j}= \overline{TPI} }_{j}+{\varepsilon}_{TPI,i,j}$$

and

3 $${{\%IA}_{i,j}= \overline{\%IA} }_{j}+ {\varepsilon}_{\mathrm{\%}IA,i,j}$$

where ${\varepsilon}_{TPI,i,j}$ and ${\varepsilon}_{\%IA,i,j}$ were independently randomly sampled from the normal distributions $\mathcal{N}(0,{{SE}_{TPI,j}}^{2})$ and $\mathcal{N}(0,{{SE}_{\%IA,j}}^{2})$, respectively. With each iteration *i*, a least-squares fit was performed based on the 5 random data points that yielded parameter values. For each source organ, the iterative process was repeated $k=\mathrm{10,000}$ times, and each parameter was determined as the mean of all iteration results.

The uncertainties in injected activity, PET quantification, and organ segmentation were not propagated into ${SE}_{\%IA,j}$, and were thus also excluded from the individual TAC fits.

### Dosimetry

The TIACs for all source organs were calculated and plotted in RStudio. Absorbed doses and effective dose were calculated in IDAC-Dose 2.1 [35]. Included source organs in IDAC-Dose 2.1 were blood, gallbladder contents, heart wall, kidneys, liver, red (active) bone marrow, salivary glands, spleen, thyroid, total body (all activity), and urinary bladder contents. Calculation of TIACs was performed with numerical trapezoidal integration (*Δ t=2.45* s) of TACs over 10 half-lives of ^11^C $t=\left[0, 3.4\right] \mathrm{h}$ (approximately 0.1% of total injected activity remaining). The fitted TACs were extrapolated beyond the time of imaging except in source organs where the accumulation rate was faster than the physical decay rate of ^11^C. Those source organs were assumed to be saturated after imaging with only physical decay as elimination mechanism (decay tail) [36].

No activity left the body during acquisition; hence, the TIAC of the whole-body was determined as $1 /{\lambda}_{physical}$. The TIAC of the remainder of the body was determined in IDAC-Dose 2.1 with the source organ “Total Body (all activity)” where the software subtracted the measured source organ TIACs from the Total Body TIAC.

For individual TAC fits, the mean and standard deviation (SD) were determined for source organ TIACs, absorbed doses, and effective dose. For parametric bootstrap TAC fits, the mean, SE, and 95% CI were determined for source organ TIACs, absorbed doses, and effective dose. To calculate the lower and upper 95% CI limits of absorbed doses and effective dose, the corresponding lower and upper 95% CI limits of all source organ TIACs were entered into IDAC-Dose 2.1. The SE values of absorbed doses and effective dose were calculated in IDAC-Dose 2.1 as the 95% CI limits for the respective quantities. Both the 95% CI and SE calculations of absorbed doses and effective dose yielded overestimates of the uncertainties because the method applied all extreme values simultaneously (assumes full correlation between source organ TIACs).

To investigate whether the parametric bootstrap method could estimate the mean and the variability in the sampled cohort in TIACs and effective dose, two ratios were calculated. 1) The ratios of the means (*individual TAC fit/parametric bootstrap TAC fit*) were calculated with an expected value of 1 (mean individual = mean parametric bootstrap). 2) The ratios of *SD/SE* (*individual TAC fit/parametric bootstrap TAC fit*) were calculated with an expected value of $\sqrt{n}$ where *n* is the cohort size, as expected under the central limit theorem.

## Results

### Participants

Ten study participants with metastasized malignancies included in the clinical trial were included in this study (8 women, 2 men) with primary malignancy from breast cancer (n = 5), ovarian cancer (n = 3), and prostate cancer (n = 2). Two participants were cholecystectomized. All participants were PARPi naïve. Basic participant information is found in Table 1.

**Table 1:** Study participant summary

Participant	Primary malignancy	Age (y)	Height (m)	Body weight (kg)	Administered activity per unit body weight (MBq/kg)	Injected protein mass (μg)	Effective dose ICRP 103 (μSv/MBq)
1	Breast	61	1.65	53.6	5.8	2.22	5.34
2	Breast	67	1.69	80.8	5.7	3.50	4.63
3	Breast	65	1.71	73.3	6.0	1.95	4.55
4	Breast	67	1.65	61.7	5.8	0.83	4.66
5	Breast	55	1.60	83.3	5.8	0.74	5.04
6	Ovarian	69	1.58	51.3	5.8	0.63	4.58
7	Ovarian	70	1.78	56.9	3.6	10.09	5.43
8	Ovarian	55	1.74	72.9	5.5	1.26	5.22
**Female Median, (Range)**		66 (55–70)	1.67 (1.58–1.78)	67.3 (51.3–83.3)	5.8 (3.6–6.0)	1.61 (0.63–10.09)	4.85 (4.55–5.43)
9	Prostate	81	1.72	82.2	5.7	0.82	5.06
10	Prostate	80	1.82	85.1	5.7	0.53	5.10
**Male Median (Range)**		81 (80–81)	1.77 (1.72–1.82)	83.7 (82.2–85.1)	5.7 (5.7–5.7)	0.68 (0.53–0.82)	5.08 (5.06–5.1)

^*^Cholecystectomized

Basic study participant information as well as: Primary malignancy, administrated activity per unitbody weight, injected protein mass, and individual TAC fit effective dose results

**Table 2:** Applied models for decay-corrected TAC fits

Biodistribution Model	Equation	Organs
**Equation 4** Mono-exponential accumulation	$A\left(t\right)={A}_{0}{e}^{\frac{\mathrm{l}\mathrm{n}\left(2\right)}{{t}_{up}}t}$	Gallbladder contents
**Equation 5** Bi-exponential 1 uptake 1 retention	$A\left(t\right)={A}_{\mathrm{m}\mathrm{a}\mathrm{x}}\left({e}^{-\frac{\mathrm{l}\mathrm{n}\left(2\right)}{{t}_{ret}}t}-{e}^{-\frac{\mathrm{l}\mathrm{n}\left(2\right)}{{t}_{up}}t}\right)$	Liver
**Equation 6** Bi-exponential direct uptake and 2 retentions	${A\left(t\right)=A}_{tot}\left({f}_{fast}{e}^{-\frac{\mathrm{l}\mathrm{n}\left(2\right)}{{t}_{ret,fast}}t}+\left(1-{f}_{fast}\right){e}^{-\frac{\mathrm{l}\mathrm{n}\left(2\right)}{{t}_{ret,slow}}t}\right)$	Blood (Aorta), heart wall, and kidneys
**Equation 7** Mono-exponential direct uptake 1 retention with baseline	$A\left(t\right)={A}_{wash}{e}^{-\frac{\mathrm{l}\mathrm{n}\left(2\right)}{{t}_{ret}}t}+{A}_{baseline}$	Lungs and thyroid
**Equation 8** Linear accumulation	$A\left(t\right)={r}_{accum}t+{A}_{0}$	Red (active) bone marrow, salivary glands, spleen, and urinary bladder contents

In all equations*t*is time.

**Equation 4: **${A}_{0}$is the initial %IA at*t=0*,${t}_{up}$is the biological accumulation half-life.

**Equation 5: **${A}_{max}$is the maximum %IA, and${t}_{up}$and${t}_{ret}$are the biological accumulation and retention half-lives respectively.

**Equation 6: **${{{A}}}_{{{tot}}}$is the total %IA at*t=0*,${f}_{fast}$is the fraction of${A}_{tot}$that is described by the fast retention compartment,${t}_{ret,fast}$and${t}_{ret,slow}$are fast and slow biological retention half-lives respectively.

**Equation 7: **${A}_{wash}$is the %IA that rapidly is washed out,${t}_{ret}$is biological retention half-life of${A}_{wash}$, and${A}_{basline}$is the baseline activity level.

**Equation 8: **${r}_{accum}$is the rate of accumulation, and${A}_{0}$is the initial %IA at*t=0*.

### Safety

The radiopharmaceutical was well tolerated by the study participants. No changes in vital signs were observed during the examination, and no AE related to [^11^C]AZ14193391 was reported within 24 h of injection. One study participant received an injection with a protein mass greater than 10 µg, which was reported and the incident did not yield any AEs.

### Biodistribution

**Fig. 1:**
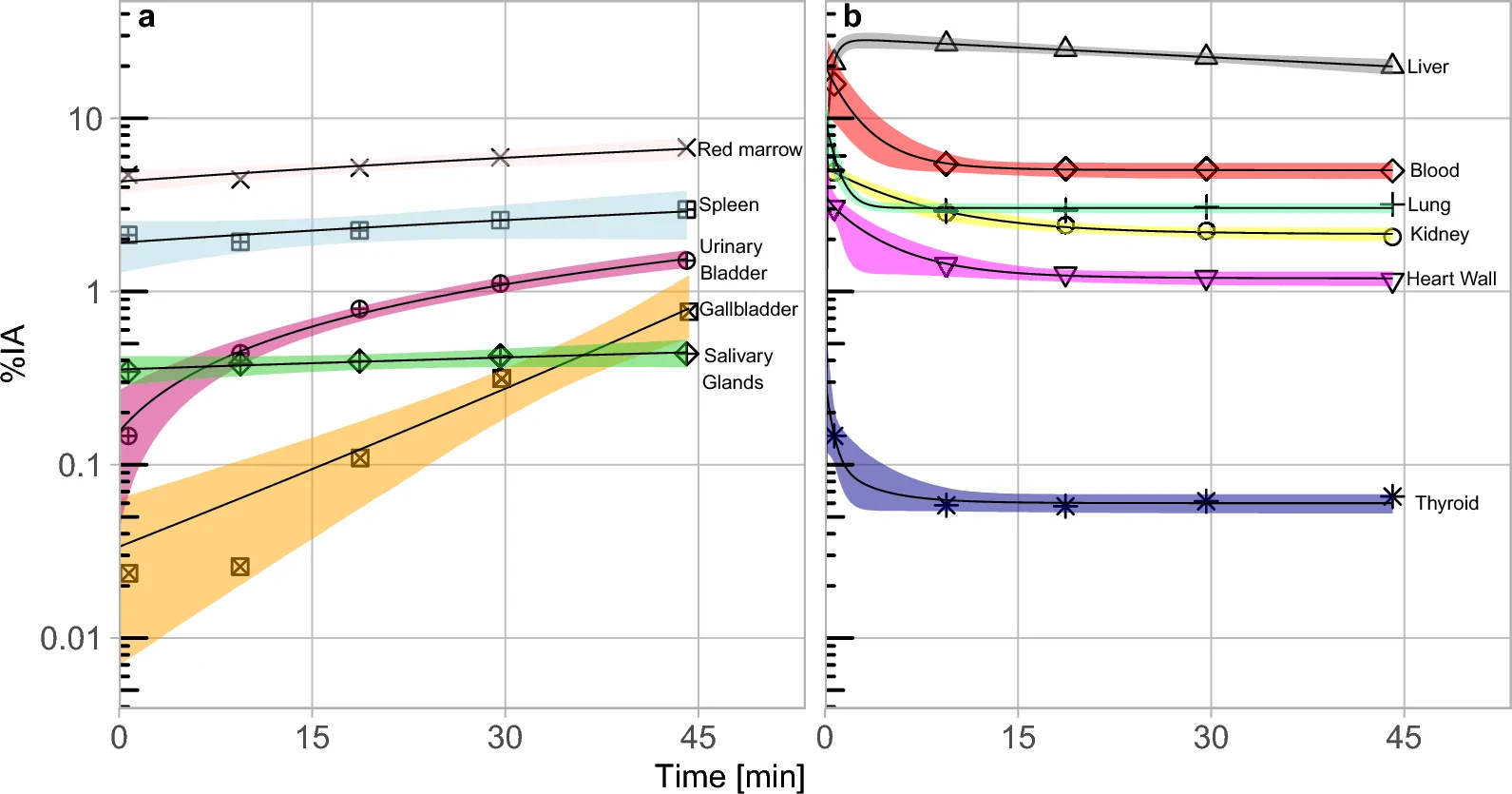
Parametric bootstrap-fitted decay-corrected TACs of the PARP1-selective radiopharmaceutical [^11^C]AZ14193391. Organs were dichotomised by measured response: a) accumulation or b) clearance of [^11^C]AZ14193391. Markers: mean %IA of each time-bin. Highlighted area: 95% CI of the fit. x-axis: time [min]; y-axis: percentage of injected activity [%IA], log-scale

**Fig. 2:**
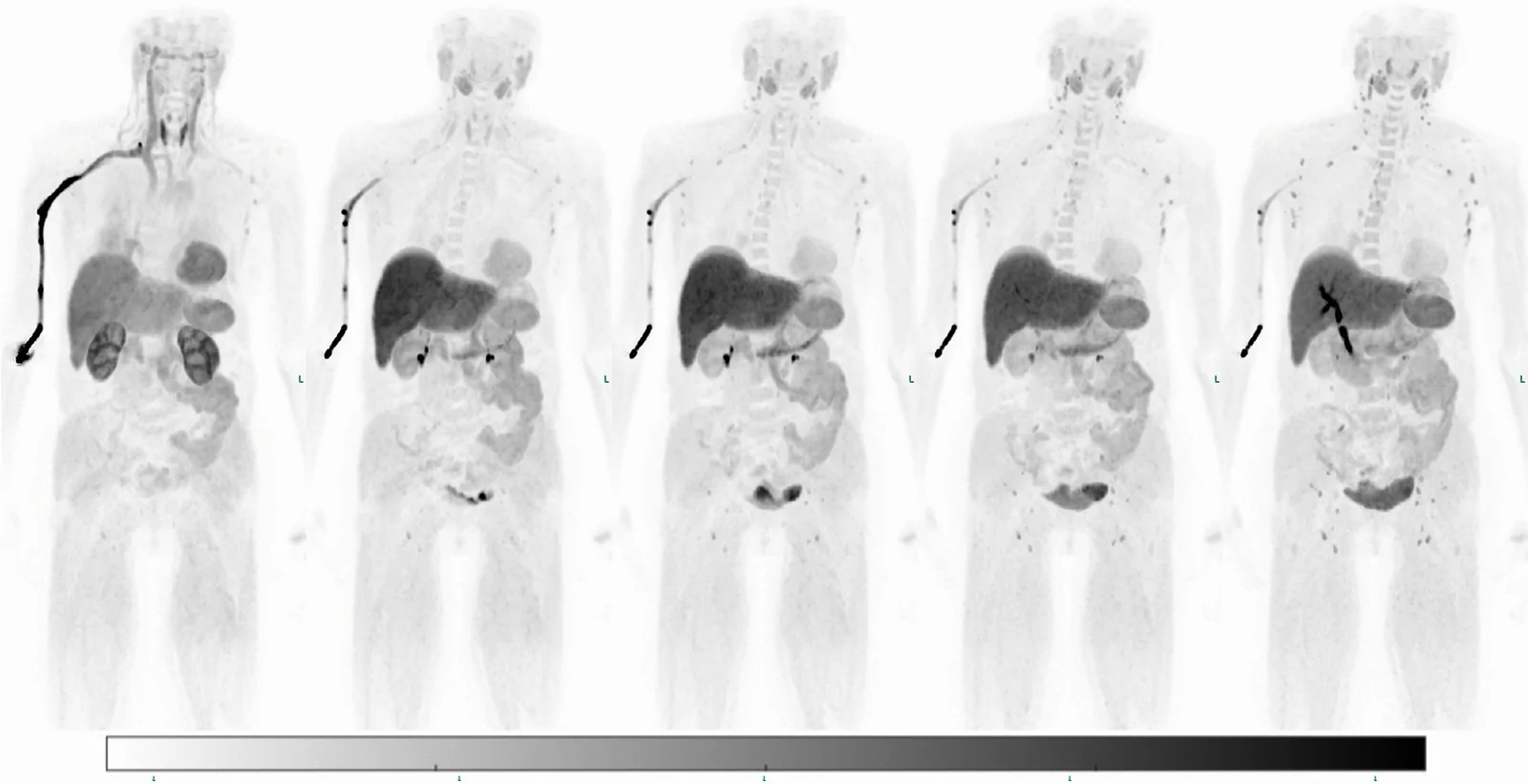
MIP images of a representative participant with ovarian cancer imaged with a ^11^C-PARP1-selective radiopharmaceutical [^11^C]AZ14193391. Acquired PET images with a GE Discovery MI PET/CT. Start of acquisition of the five sequential PET acquisitions were (from left) approximately 0, 10, 20, 30, and 45 min post injection of [^11^C]AZ14193391. All images have equal window settings (SUV scale range 0 – 17.5). Visual evaluation of PET images shows high activity concentration in the gastrointestinal tract, liver, kidneys, red marrow, pancreas, salivary glands, and spleen

Parametric bootstrap TACs, illustrated in Fig. 1, indicate that the source organs with highest activity were in the blood, kidneys, liver, lungs, RM, and spleen. Visual evaluation of PET images showed increased signal compared to background tissue (adipose and muscle) in the gastrointestinal (GI) tract, liver, kidneys, RM, pancreas, salivary glands, and spleen. In Fig. 2, maximum intensity projection (MIP) PET images of a participant from the ovarian cancer cohort acquired at five time points are shown.

In Table 2, the five different models found to fit the eleven source organ TACs are listed along with parameter explanations.

Parametric bootstrap decay-corrected TAC fit results of source organs are summarised in Table 3 and the decay-corrected source organs TACs are illustrated in Fig. 1. The individual decay-corrected source organs TACs are illustrated in Supplementary Figure S1. In source organs RM, thyroid, salivary glands, spleen one or several study participants activity measurements significantly deviated from the TAC models applied in parametric bootstrap TAC fits. Those individual fits were either set to a constant value or applied different TAC model.

**Table 3:** Parametric bootstrap-fit results of decay-corrected TACs

**Equation 4** Mono-exponential accumulation	${A}_{0}$[%IA] Mean (SE)	${t}_{up}$[h] Mean (SE)		
*Gallbladder contents*	3.36E-02 (1.45E-02)	1.63E-01 (3.08E-02)		
**Equation 5** Bi-exponential 1 uptake 1 retention	${\overline{A} }_{max}$[%IA] Mean (SE)	${\overline{t} }_{up}$[h] Mean (SE)	${\overline{t} }_{ret}$[h] Mean (SE)	
*Liver*	2.92E + 01 (1.76E + 00)	6.48E-03 (1.60E-03)	1.44E + 00 (4.64E-01)	
**Equation 6** Bi-exponential direct uptake and 2 retentions	${\overline{A} }_{tot}$[%IA] Mean (SE)	${\overline{f} }_{fast}$ Mean (SE)	${\overline{t} }_{ret,fast}$[h] Mean (SE)	${\overline{{{t}}} }_{{{ret}},{{slow}}}$[h] Mean (SE)
*Blood*	2.01E + 01 (5.55E + 00)	7.30E-01 (8.17E-02)	3.07E-02 (1.30E-02)	120*
*Heart wall*	3.62E + 00 (1.02E + 00)	6.56E-01 (5.44E-02)	4.98E-02 (1.77E-02)	120*
*Kidney*	5.19E + 00 (3.04E-01)	5.84E-01 (2.74E-02)	7.93E-02 (1.94E-02)	120*
**Equation 7** Mono-exponential direct uptake 1 retention with baseline	${\overline{A} }_{wash}$[%IA] Mean (SE)	${\overline{t} }_{ret}$[h] Mean (SE)	${\overline{A} }_{baseline}$[%IA] Mean (SE)	
*Lung*	7.01E + 00 (1.89E + 00)	0.01*	3.03E + 00 (9.78E-02)	
*Thyroid*^*‡¶†*^	2.51E-01 (1.60E-01)	1.93E-02 (1.78E-02)	6.00E-02 (3.82E-03)	
**Equation 8** Linear accumulation	${\overline{r} }_{accum}$[%IA/h] Mean (SE)	${\overline{A} }_{0}$[%IA] Mean (SE)		
*Red (active) bone marrow*	3.20E + 00 (9.39E-01)	4.31E + 00 (3.32E-01)		
*Salivary glands*^*‡||*^	1.23E-01 (8.69E-02)	3.55E-01 (3.44E-02)		
*Spleen*^*‡||*^	1.34E + 00 (9.15E-01)	1.91E + 00 (3.22E-01)		
*Urinary bladder*	1.89E + 00 (1.75E-01)	1.55E-01 (5.79E-02)		

^*^Fixed value

^‡^One or several of individual TAC fits used different TAC model from the parametric bootstrap fit

^¶^Linear fit (1 of 10 thyroid fits)

^†^Non-fitted (trapezoidal integration) (1 of 10 thyroid fits)

^||^Constant value (mean %IA) due to non-significant slope (*p* > 0.05) (6 of 10 salivary glands fit and 3 of 10 spleen fits)

### Dosimetry

Extrapolation of TACs for integration beyond the imaging time was feasible for all organs except the gallbladder contents, where the accumulation rate of [^11^C]AZ14193391 was greater than the decay rate of ^11^C. Therefore, the gallbladder TIACs were calculated with a decay tail post-imaging.

The median ratio of the TIACs integrated from the time of the first to the last PET acquisition to the total TIACs (TIAC imaging/TIAC total) showed that 73% (range 42–83%) of the TIACs were within the timespan of the PET acquisitions.

**Fig. 3:**
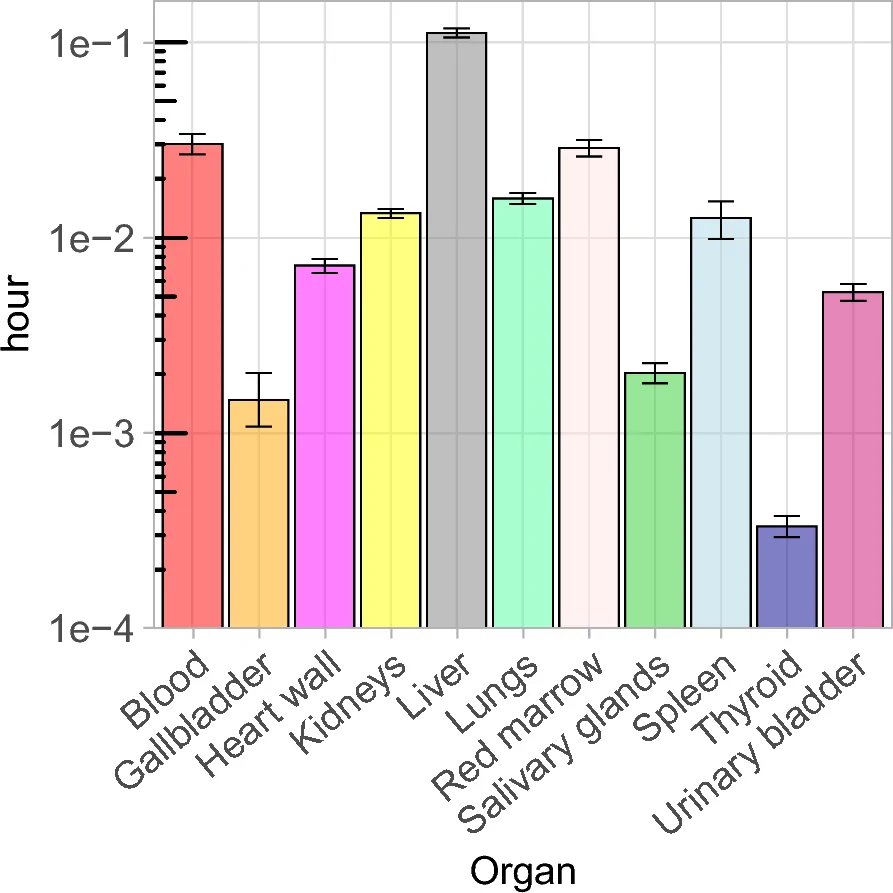
Parametric bootstrap TIAC results of the PARP1-selective radiopharmaceutical [^11^C]AZ14193391. Error bars (95% CI) (log-scale). TIAC calculated with trapezoidal integration (range 0–3.4 h) *Δ t=2.45* s. Colour coding matches Fig. 1

In Fig. 3, the parametric bootstrap TIAC results with error bars representing 95% CI for respective source organ are illustrated. Blood, liver, and RM were the source organs with the largest TIACs. The median ratio of *SD/SE* for source organ TIACs was 5.74 (range 2.85–6.75), with 10 study participants, the expected value was $\sqrt{10}\approx 3.16$. This suggests that the parametric bootstrap method could not estimate the variability of TIACs in the sampled cohort. The exception was the gallbladder TIAC where only 8 of 10 study participants had a gallbladder and the ratio of *SD/SE* was $2.85\approx \sqrt{8}$.

**Table 4:** Source organ TIACs, absorbed dose to organs and effective dose results of [11C]AZ141

Organs	Individual TAC method				Parametric bootstrap TAC method		
	TIAC (SD) [h]	D_male_ (SD) [μGy/MBq]	D_female_ (SD) [μGy/MBq]	D_average_	TIAC (SE) [h]	D_male_ (95%CI) [μGy/MBq]	D_female_ (95%CI) [μGy/MBq]
Blood	3.01E-02 (8.37E-03)				3.02E-02 (1.90E-03)		
Gallbladder wall*	1.51E-03 (7.15E-04)	8.40 (0.07)	11.36 (1.44)	10.77 (1.75)	1.48E-03 (2.50E-04)	9.50 (8.88–10.2)	11.4 (10.6–12.3)
Heart wall	7.22E-03(1.66E-03)	7.95 (0.23)	9.49 (1.50)	9.18 (1.48)	7.22E-03 (3.07E-04)	7.53 (6.96–8.07)	9.64 (8.90–10.4)
Kidneys	1.33E-02 (2.37E-03)	10.80 (0.10)	13.54(2.06)	12.99 (2.14)	1.33E-02 (3.51E-04)	11.2 (10.6–11.8)	13.4 (12.7–14.1)
Liver	1.11E-01 (1.87E-02)	15.60 (1.40)	21.04 (3.12)	19.95 (3.59)	1.11E-01 (3.02E-03)	16.5 (15.6–17.4)	20.7 (19.6–21.9)
Lung	1.57E-02 (3.28E-03)	5.90 (0.02)	6.93 (0.77)	6.73 (0.81)	1.58E-02 (5.17E-04)	5.75 (5.48–6.01)	7.00 (6.67–7.33)
Red marrow	2.86E-02 (7.10E-03)	5.18 (0.12)	6.17 (0.78)	5.97 (0.80)	2.88E-02 (1.43E-03)	4.92 (4.65–5.20)	6.29 (5.90–6.69)
Salivary glands	1.99e-03(6.99e-04)	4.66 (0.49)	7.76 (2.15)	7.14 (2.30)	2.03E-03 (1.22E-04)	6.04 (5.45–6.63)	7.49 (6.76–8.23)
Spleen	1.25e-02 (7.98e-03)	27.45 (7.75)	16.46 (7.42)	18.66 (8.68)	1.26E-02 (1.38E-03)	16.4 (13.3–19.6)	19.8 (16.0–23.6)
Thyroid	3.36E-04 (1.29E-04)	3.65 (0.70)	5.88 (1.29)	5.43 (1.49)	3.33E-04 (2.15E-05)	4.66 (4.26–5.09)	5.53 (5.05–6.05)
Urinary bladder wall*	5.39E-03 (1.33E-03)	3.30 (0.01)	3.53 (0.68)	3.49 (0.62)	5.26E-03 (2.66E-04)	3.31 (3.29–3.34)	3.63 (3.61–3.65)

^*^TIAC measured in organ contents

TIAC calculations were performed with two TAC fit methods: 1) individual (mean TIAC(SD)) and 2) parametric bootstrap (mean (TIAC (SE)). The 95 % CI were estimated by

The organs exposed to the highest absorbed dose per 1 MBq of injected activity were liver, spleen, and kidneys (Table 4). When organ absorbed doses were compared between male and female participants, the parametric bootstrap method showed non-overlapping 95% CI in 8 out of 10 source organs. With the ICRP Publication 103 tissue weighting factors applied, the mean effective dose per injected activity of [^11^C]AZ14193391 was estimated to be 5.0 (SD = 0.3) µSv/MBq with the individual TAC method and 5.0 (SE = 0.1) µSv/MBq with the parametric bootstrap TAC method (see Table 5). This corresponds to an effective dose of 2.0 mSv for a person weighing 70 kg injected with 5.7 MBq/kg (400 MBq) of the radiopharmaceutical, as was performed in this study. The ratio of *SD/SE* for effective dose was approximately $3=\sqrt{9}<\sqrt{10}$, which suggests that the parametric bootstrap method did estimate the variability of effective doses in the sampled cohort (Table 6).

**Table 5:** Effective dose results

***TAC fit method***	*Effective dose 103* [µSv/MBq]	95% CI [µSv/MBq]
Individual (SD)	5.0 (0.3)	
Parametric bootstrap (SE)	5.0 (0.1)	(4.8–5.2)

The effective dose results of the PARP1-selective radiopharmaceutical [^11^C]AZ14193391. Effective dose (ED) was determined with two TAC fit methods: Individual (estimates sampled cohort variability: ED (SD)) and parametric bootstrap (estimates the precision of the sampled cohort mean: ED (SE)). The 95% CI was estimated by inserting the CI lower or CI upper TIACs of respective source organ assuming full correlation of TIACs.

**Table 6:** SD/SE-ratios

***Ratio***	TIAC Median (Range)	ED
SD/SE-ratio	5.74 (2.85–6.75)	3.0
Ratio to expected value^†^	1.82 (0.90–2.13)	0.95

^†^ Expected value = √10 = 3.16

Calculated ratios of individual TAC fit results to parametric bootstrap TAC fit results. Ratio to expected value indicates how well the parametric bootstrap method recovers the variability of the sampled cohort, where 1 indicates perfect recovery

## Discussion

[^11^C]AZ14193391 is a novel PARP1-selective radiopharmaceutical that has previously been demonstrated to have similar binding affinity to olaparib and palacaparib and the ability to penetrate the blood–brain barrier [26]. In this study, we investigated the safety, determined the biodistribution in healthy organs, the absorbed doses, and effective dose in patients with metastatic solid malignancies.

Biodistribution curves indicate that [^11^C]AZ14193391 was excreted mainly through the hepatobiliary pathway and showed that there was an accumulation in the GI tract, RM, salivary glands, and spleen. These visual assessments correspond well with the biodistribution measurements in the gallbladder, RM, salivary glands, and spleen, where [^11^C]AZ14193391 accumulates during the first hour p.i. In the gallbladder, RM, salivary glands, and spleen, the peak ${A}_{C}$ of [^11^C]AZ14193391 was not encountered during the acquisitions (see Fig. 1). Therefore, the ability to fully estimate the biodistribution in these organs was limited.

The biodistribution curves of the heart wall, lungs, and thyroid seen in Fig. 1 showed that [^11^C]AZ14193391 reached steady state approximately 15 min p.i., as did the blood curve. This suggests that there is low or no accumulation of [^11^C]AZ14193391 in these organs and the measured activity primarily reflected blood activity.

Due to the function of the gallbladder, which empties in response to food intake or periodically when fasting, the decay tail assumption could either under- or overestimate its TIAC. The gallbladder contents could continue accumulation post-imaging and/or be emptied before the activity reaches a negligible level. This uncertainty was also relevant in the urinary bladder contents TIAC, where continued accumulation post-imaging without voiding was assumed. The remainder TIAC was affected by this uncertainty as well, as it varies with source organs TIACs.

Currently, there are several diagnostic PARP-selective PET radiopharmaceuticals in different stages of development. [^18^F]fluorthanatrace (FTT) is a rucaparib-analogue and is the most-studied PET radiopharmaceutical with a first-in-human dosimetry study that reported the highest absorbed doses in the spleen, pancreas, and liver [37]. However, direct comparison of [^18^F]FTT to [^11^C]AZ14193391 should be performed with caution since the two are based on different carrier molecules and different isotopes.

[^18^F]PARPi is an olaparib-analogue PET radiopharmaceutical that has been demonstrated in mice and 7 human participants to cross the blood–brain barrier and bind to PARP1 in malignancies [38]. Schöder et al. published dosimetry for [^18^F]PARPi with a mean effective dose of 14 µSv/MBq and reported the highest absorbed doses in the gallbladder wall, kidneys, and liver [39]. However, it is difficult to directly compare the results as different isotopes, TAC fit methods, and effective dose calculations were used. For [^18^F]FTT and [^18^F]PARPi, like [^11^C]AZ14193391, the kidneys, liver, and spleen received the highest absorbed doses. Zanotti-Fregonara and Innis summarised 21 ^11^C dosimetry studies, reporting a mean effective dose of 5.1 (SD = 2.2) µSv/MBq, suggesting our result 5.0 (SD = 0.3) is comparable to other published values [40].

When the individual and parametric bootstrap TAC fit results were compared, both methods showed equivalent TIAC means but with a median ratio of *SD/SE* of 5.74. The exception was the gallbladder contents, whose results agreed with the equality $SD/SE=\sqrt{n}$. This was probably because 58% of the TIAC was from post-imaging and thus the majority of its TIAC was not participant-dependent. Like the TIACs, the mean effective dose ratio was 1, but the ratio of *SD/SE* was reduced to 3. This suggests that the parametric bootstrap method may be used to estimate the variability in population effective doses, which could render the individual TAC fit method redundant for diagnostic radiopharmaceuticals. However, the parametric bootstrap method did not correctly estimate the variability of individual TIACs in the sampled cohort and should thus be used with caution. Further investigation is needed to evaluate the parametric bootstrap TAC method’s potential to estimate the population variability of TIACs and effective dose for cohorts of different sizes and for other radioisotopes before it could replace the individual TAC fit method.

The lack of high-frequency PET acquisitions during the first 5 min p.i. led most source organs to be fitted with a direct uptake TAC model. The direct-uptake assumption is generally invalid and overestimates TIACs when integrated from *t=0*.

The relatively short half-life of ^11^C (20.37 min [41]) limited the ability to measure organs with low ${A}_{C}$. Such organs became susceptible to noise in the later PET acquisitions. The short acquisition window (1 h) limited the ability to measure biodistribution in organs with a slow accumulation phase (gallbladder contents, RM, salivary glands, spleen, and urinary bladder contents) and the extrapolated portion of TIAC had a median of 31% (range 27–58%), which is the TIAC portion with the largest unquantified uncertainty. The corresponding proportion in organs with clearance was 18% (range 16–21%). However, for the dosimetry results, the TIACs of organs with accumulation were likely overestimated as the extrapolation assumed infinite accumulation except for the gallbladder contents where the decay tail assumption was applied.

The magnitude of the errors in injected activity measurements and in PET quantification have been estimated in the literature as 5% [42] and 10% [43], respectively. The error of activity in segmented organs is presumably low as the segments were expanded to cover the full PET signal, which recaptures spill-out. The approximate minimum total additional propagated error is 11% in the total activity measurements.

Furthermore, the tumour sink effect, which has been demonstrated to affect radiopharmaceutical accumulation in healthy organs [44, 45], was not quantitatively analysed. The neglect of tumour sink effect analysis could lead to an underestimation of the dosimetry when TIACs estimated from cancer subjects are applied to healthy subjects. The tumour sink effect is not directly proportional to the tumour burden, but rather to the fraction of the administered radiopharmaceutical that a tumour sequesters. Gafita et al. defined the tumour sink effect as relevant if the decrease in healthy organ uptake was ≥ 30%, which was found in subjects with a [^68^Ga]Ga-PSMA-11 positive tumour volume ≥ 1,355 cm^3^ [45]. In our study cohort, there was a wide range of tumour burden in terms of tumour stage. However, PET images showed only an increased ${A}_{C}$ in a few small lesions, and thus a risk of a tumour sink effect on the dosimetry is expected to be low.

## Conclusion

Our results suggest that PET imaging with [^11^C]AZ14193391 is feasible and safe. The associated effective dose is low (5.0 µSv/MBq) with the highest absorbed doses measured in the liver (16.5 µGy/MBq), spleen (16.4 µGy/MBq), and kidneys (11.2 µGy/MBq). Furthermore, two methods were used to determine organ absorbed doses and effective dose, namely the individual TAC method and the parametric bootstrap TAC method. The results of both methods yielded equivalent means, and the parametric bootstrap estimated the population variability in the effective dose, but not in the TIACs.

This study paves the way for further investigation of the potential clinical use of [^11^C]AZ14193391 as a tool to identify eligible patients for PARPi treatment.

## Supplementary Information

Additional file 1: Supplementary Figure S1. Individual decay-corrected TAC fit results. Solid lines are individual fits where the same biodistribution model as the parametric bootstrap fit was applied. Dashed lines are individual fits where a different biodistribution model from the parametric bootstrap fit was applied. The specific biodistribution models applied are listed in Table 3.

## Data Availability

The datasets generated and analysed during the current study are available from the corresponding author on reasonable request.

## Abbreviations

AE: Adverse event
$${A}_{C}$$: Activity concentration
*BRCA1/2*: Breast cancer gene 1 or 2
CT: Computed tomography
FTT: Fluorthanatrace
HRD: Homologous recombination deficiency
*HER2*: Human epidermal growth factor receptor 2
PARPi: PARP inhibitor
PVE: Partial volume effect
*%IA*: Percentage of injected activity
PARP: Poly (ADP-ribose) polymerase
PARP1: Poly (ADP-ribose) polymerase-1
PET: Positron emission tomography
p.i.: Post injection
RM: Red bone marrow
SD: Standard deviation
SE: Standard error
*TPI*: Time post-injection
TAC: Time-activity curve
TIAC: Time-integrated activity coefficient
$${A}_{tot}$$: Total activity
