# Supplementary material for: Biodistribution and dosimetry of the carbon-11 labelled PARP1-selective PET radiopharmaceutical [11C]AZ14193391
Source: EJNMMI Res. 2026 Aug 19;16:131. doi: 10.1186/s13550-026-01481-1 (PMC13504008; doi:10.1186/s13550-026-01481-1)
Supplement: Supplementary file 1 [file 13550_2026_1481_MOESM1_ESM.pdf]

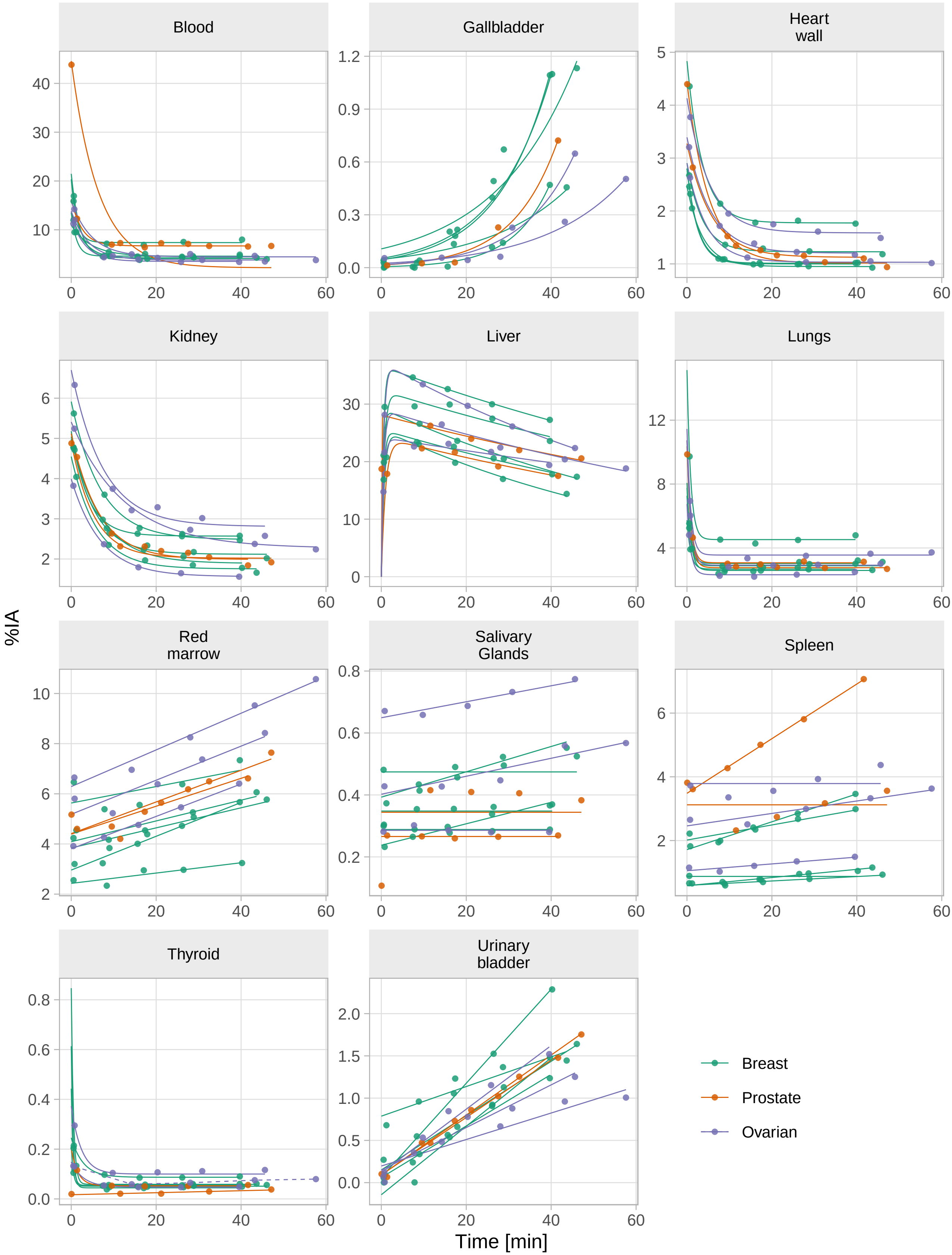

Blood
Gallbladder
Heart wall
Kidney
Liver
Lungs
Red marrow
Salivary Glands
Spleen
Thyroid
Urinary bladder
%IA
Time [min]
Breast
Prostate
Ovarian
